# Adeno-associated virus gene delivery of broadly neutralizing antibodies as prevention and therapy against HIV-1

**DOI:** 10.1186/s12977-018-0449-7

**Published:** 2018-10-01

**Authors:** Allen Lin, Alejandro B. Balazs

**Affiliations:** 1 Ragon Institute of MGH, MIT and Harvard, Cambridge, MA 02139 USA; 2 Department of Systems Biology, Harvard University, Boston, MA 02115 USA

**Keywords:** Vectored delivery, Antibody gene transfer, AAV, HIV-1, bNAb, Clinical trials, Animal models

## Abstract

Vectored gene delivery of HIV-1 broadly neutralizing antibodies (bNAbs) using recombinant adeno-associated virus (rAAV) is a promising alternative to conventional vaccines for preventing new HIV-1 infections and for therapeutically suppressing established HIV-1 infections. Passive infusion of single bNAbs has already shown promise in initial clinical trials to temporarily decrease HIV-1 load in viremic patients, and to delay viral rebound from latent reservoirs in suppressed patients during analytical treatment interruptions of antiretroviral therapy. Long-term, continuous, systemic expression of such bNAbs could be achieved with a single injection of rAAV encoding antibody genes into muscle tissue, which would bypass the challenges of eliciting such bNAbs through traditional vaccination in naïve patients, and of life-long repeated passive transfers of such biologics for therapy. rAAV delivery of single bNAbs has already demonstrated protection from repeated HIV-1 vaginal challenge in humanized mouse models, and phase I clinical trials of this approach are underway. Selection of which individual, or combination of, bNAbs to deliver to counter pre-existing resistance and the rise of escape mutations in the virus remains a challenge, and such choices may differ depending on use of this technology for prevention versus therapy.

## Background

HIV-1 remains a significant contributor to the global burden of disease. In 2016, 1.8 million individuals were newly infected with HIV-1, and more than 36 million individuals were living with HIV-1, of whom only 44% were virally suppressed with antiretroviral therapy (ART) [1]. The need for daily dosing of ARTs remains a challenge for their effective use for both viral suppression as well as pre-exposure prophylaxis of HIV-1. Whether due to lack of drug access, stigma, inability, or drug-drug interactions, failure to maintain drug pressure in the body can result in breakthrough infection or drug-resistant viral rebound. Long-term, continuous, systemic expression of anti-HIV-1 antibodies by a single administration of recombinant adeno-associated viruses (rAAV) may be an alternative to ARTs.

This review will summarize advances in using recombinant AAV (rAAV) for gene transfer, and describe broadly neutralizing antibodies (bNAbs) against HIV-1 and the results of recently completed clinical trials that passively transfer these bNAbs into individuals living with HIV-1. It also describes recent progress of vectored delivery of bNAbs for long-lasting expression in humanized mouse models, macaque models, and in ongoing clinical trials, and concludes with the challenges faced in deciding which bNAbs to deliver.

## Main text

### Recombinant adeno-associated viruses (rAAV) for gene transfer

AAVs have long been contemplated as attractive vectors for use in gene transfer [2]. AAV is a replication-defective 20–25 nm *Parvoviridae* virus consisting of a non-enveloped, icosahedral protein shell (capsid) surrounding one copy of a linear single-stranded DNA genome. Initially found in 1965 as a contaminant of adenovirus preparations [3], AAV can only replicate within cells in the presence of helper functions provided by viruses such as adenovirus or herpesvirus. The 4.7 kb AAV genome encodes for *rep* and *cap* in between two palindromic 145 bp inverted terminal repeats (ITRs). These ITRs self-anneal into T-shaped hairpin structures [4]. *rep* is translated into four non-structural proteins for packaging and replication and *cap* into three structural capsid proteins that protect the genome and modulate cell binding and trafficking. In addition, a recently discovered alternative open-reading frame in *cap* encodes for assembly-activating protein, which is necessary for capsid assembly in certain AAV serotypes [5]. Thirteen serotypes of AAV (named AAV1-13) have been discovered to date, and these serotypes differ in tissue tropisms, transduction efficiencies, and expression levels dependent on their viral capsid sequence [6]. Screening in humans and nonhuman primates and ancestral sequence reconstruction have identified numerous additional infectious capsids that are variants of the 13 representative serotypes [7–9].

The ITRs are the only sequence elements required *in cis* for packing of the genome into the capsid and for replication. Thus, recombinant AAV (rAAV) vectors used for gene transfer need only consist of an expression cassette encoding a promoter and transgene placed between the ITRs, in lieu of *rep* and *cap.* Helper functions of *rep* and *cap* are supplied *in trans* via a separate plasmid, co-transfected during production, and thus no viral genes are encoded by rAAV. The serotype choice for *cap* provided *in trans* dictates the identity of the capsid shell of the recombinant vector and thus which tissues are preferentially infected by rAAV. Given the importance of *cap* in modulating tissue tropism and possibly immunogenicity [10, 11], numerous efforts are underway to engineer *cap* for greater specificity and desirable activities [12].

AAVs have no apparent pathogenicity, as they are not known to be associated with any human disease [13]. Natural AAV infection occuring without helper virus functions can enter a latent phase and integrate site-specifically into the AAVS1 site on the 19th chromosome in humans, in a process that requires proteins encoded by *rep* [14–16]. Because rAAV vectors do not encode *rep*, their genomes persist as extrachromosomal episomal concatemers that rarely integrate into the chromosome [17–19]. Despite the episomal nature of rAAV, a single intramuscular injection of rAAV has been shown to maintain transgene expression for a number of years in a variety of animal models including humans [20–23], in one case enabling detection of rAAV transgene expression in a patient over 10 years after administration [24].

There are several general considerations to using rAAV as a gene transfer vector. First, rAAV has a limited transgene carrying capacity. AAV has a genome of 4.7 kb, and rAAVs that are produced with transgenes of more than approximately 5 kb result in substantially reduced transduction efficiencies [25]. Second, transgene expression upon transduction of target tissues with single-stranded rAAV is not immediate, as the cell must first synthesize the second strand using the single-stranded DNA genomic template [26, 27]. Lastly, pre-existing immunity of individuals to AAV from natural exposure may limit the efficiency of transduction. Global seroprevalences of different AAV serotypes range from 30 to 60% [28, 29]. Even if transduction is able to occur, adaptive immune responses can severely limit transgene expression. In early gene therapy trials using AAV2 to deliver factor IX to patients with hemophilia B, factor IX expression was limited to only several months, likely due to transduced cells presenting AAV capsid peptides, which reactivated memory T cells targeting those transduced cells [30]. Subsequent trials using AAV8 were successful in stable expression of factor IX when they excluded patients with detectable anti-AAV antibodies and used the lowest dose of rAAV8 that still provided therapeutic benefit [23, 31]. Efforts to discover rare and ancestral AAV capsids and to create new capsids for which humans do not yet have an immune response are underway [9]. Since a patient who receives rAAV will likely develop immunity against the capsid upon injection, subsequently giving the same patient another rAAV with the same capsid serotype is unlikely to result in additional transgene expression.

Only two rAAV gene therapy products have been licensed to date, but many more are in clinical trials [32]. Glybera (alipogene tiparvovec) for lipoprotein lipase deficiency was the first gene therapy product licensed in Europe in 2012, in which the human lipoprotein lipase gene in an AAV1 capsid is administered via intramuscular injection. Luxturna (voretigene neparvovec) for inherited retinal dystrophy was the first gene therapy product approved by the FDA in 2017, in which the RPE65 gene in an rAAV2 vector is injected subretinally to treat blindness [33, 34]. Affordability and patient accessibility of gene therapy products remain to be determined. Priced at 1 million dollars per treatment, Glybera was withdrawn from the market by its manufacturer after 5 years [35]. Due to difficulty of convincing national reimbursers to pay for the treatment, it was only used in one patient. Luxtura has been similarly priced at $425,000 per eye [36]. To increase its acceptability, its manufacturer is seeking reimbursement only with positive outcomes. The prices of future gene therapy products will depend on the commercial outcomes of these initial products and further maturation and widespread adoption of these technologies.

### Anti-HIV-1 broadly neutralizing antibodies (bNAbs)

Gene transfer of anti-HIV-1 broadly neutralizing antibodies (bNAbs) with rAAV may be an effective method to prevent and suppress HIV-1 infection. Approximately half of chronically infected HIV-1 individuals naturally develop sera capable of neutralizing half of the diversity of HIV-1 at low to moderate titers [37]. However, only a small proportion of individuals develop bNAbs of great potency and breadth that cross-clade neutralize diverse HIV-1 strains, by binding to conserved regions of the HIV-1 envelope spike. These rare bNAbs are heavily somatically hypermutated from years of coevolution with the virus [38]. Several properties of HIV-1 envelope impede the development of such antibodies. First, a single HIV-1 virion displays only ~ 14 envelope spikes on its surface [39]. Such low-density surface protein limits the potential for avidity effects and thus may result in less BCR cross-linking for B cell activation. Second, the envelope surface is covered by shifting glycosylation sites and flexible variable loops that sterically hinder access to conserved epitopes buried deep inside the protein, and thus antibodies against HIV-1 are more likely to be strain-specific than broad [40, 41].

Nevertheless, improvements in antibody discovery techniques have resulted in the identification of new bNAbs each year [42]. Antibodies appear to bind to several preferential target regions on the HIV-1 envelope: the V1/V2 site at the trimer apex, the N332 glycan supersite near the V3 loop, the CD4 binding site, the gp120–gp41 interface, and the membrane-proximal external region (MPER) [43]. The CD4 binding site is of particular interest as it is well conserved due to the need for HIV-1 to bind to its primary receptor for infection. bNAbs that target the CD4 binding site include b12 [44], VRC01 [45, 46], 3BNC117 [47], N6 [48], and N49P7 [49]. These latter antibodies possess great breath and potency, as N49P7 neutralized 86% of a 117 multi-clade pseudovirus panel at an IC_50_ < 1 μg/ml [49], and N6 neutralized 96% of another 181 multi-clade pseudovirus panel at an IC_50_ < 1 μg/ml [48].

However, eliciting bNAbs in individuals through vaccination is likely to be difficult as a consequence of the extensive somatic hypermutation and unusually long sequence complementarity determining regions observed in many bNAb lineages. Thus, novel sequential administration of different immunogens may be needed to elicit bNAbs in patients [50]. Multiple immunogen design strategies have emerged to first stimulate bNAb germline precursors and then drive affinity maturation against bNAb target epitopes [51]. In lineage-based immunogen design, immunogens mimic natural viral evolution found in a patient who develops a bNAb, starting with the founder strain [52]. In germline-targeting immunogen design, the first immunogen seeks to engage bNAb germline precursors. For instance, eOD-GT8 is a multivalent nanoparticle, presenting a structure-based design of gp120 outer domain molecule selected through iterative random mutagenesis and yeast cell surface display [53]. Priming with this immunogen followed by more native Env-like boosts in a VRC01 germline knock-in model resulted in antibodies of intermediate VRC01 maturity [54]. Results of these approaches are promising, but guiding such maturation in diverse patient populations may be difficult due to allelic diversity at immunoglobulin loci. Given the likely difficulty of eliciting bNAbs via traditional vaccines, alternative approaches using existing bNAbs either through passive transfer or gene transfer are being explored.

### Passive transfer of bNAbs in clinical trials

**Fig. 1:**
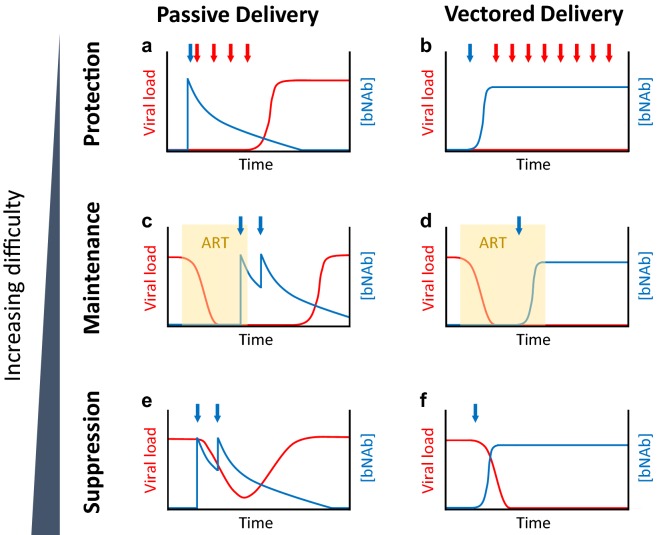
Experimental designs for in vivo efficacy testing of bNAbs, delivered passively or vectored, against HIV-1. Three designs are shown in increasing order of difficulty in achieving success. Schematics of viral load (red line) and bNAb concentration (blue line) over time are shown, and passive or vectored delivery of bNAb (blue arrows) and HIV-1 challenges (red arrows) are indicated. In these graphs, the bNAb neutralizes the HIV-1 strain, and escape mutations are not pre-existing nor emerge. HIV-1 can replicate when the bNAb is below a certain concentration. **a**, **b** Protection from HIV-1 challenge. **c**, **d** Maintenance of ART-suppressed virus in an analytical treatment interruption (ATI). ART treatment is interrupted after the desired bNAb concentration is achieved. The particular ART used may hinder second strand synthesis of rAAV, in which case the bNAb can be passively infused simultaneously with vectored delivery to maintain suppression (not shown). Viral reactivation from latent reservoirs continuously occurs, and greater viral dissemination prior to ART suppression likely increases the latency burden and frequency of reactivation events. **e**, **f** Suppression of replicating viremia. Millions to billions of viral particles are replicating and mutating when bNAb pressure is exerted, creating a selection force that advantages escape mutants. To achieve complete suppression, the bNAb will need to neutralize not just the dominant strain, but all of the existing minor strains and the potential emergent mutants in the viral quasispeices

Given the challenges of eliciting highly somatically mutated bNAbs in naïve individuals, direct administration of mature bNAbs for prevention or therapy is currently being tested in humans. Experimental designs for these clinical trials are shown in Fig. 1. Six phase I or IIa trials of passive infusion of single bNAbs (VRC01 [55, 56], 3BNC117 [57, 58], and 10-1074 [59]) into HIV-1 infected individuals have been published to date (Table 1), with many more underway or planned [60]. These studies use one of two therapy trial protocols. The first consists of administration of antibody into viremic individuals and observing the decline in viral load and time until viral rebound (Fig. 1e). The second is an analytic treatment interruption (ATI), where ART-suppressed HIV-infected individuals are given multiple sequential antibody infusions and taken off ART shortly after the first infusion (Fig. 1c). The delay in latent viral rebound is then observed. Overall, these studies have shown that the examined bNAbs have a therapeutic effect and exert selection pressure on the virus. The degree of suppression varied across antibodies and patients, depending on antibody potency and the presence of pre-existing resistance mutations in a patient. Interestingly, despite theoretical concerns over the degree of somatic hypermutation these antibodies exhibit, these bNAbs have not been found to be particularly immunogenic in humans, as anti-drug antibody (ADA) responses have not been observed in these trials. Across all of these trials, bNAb half-life was consistently shorter in HIV-1 infected individuals than in uninfected individuals, perhaps due to increased clearance of antibody-antigen immune complexes.

**Table 1:** Clinical trials of bNAbs in HIV-1 infected individuals with published results

Clinical trial	Trial design	Delivery	bNAb	Highest dose given (mg/kg)	HIV-1 infected individuals given highest dose	Dosing schedule	bNAb sensitivity prescreening	Viral response	References
NCT02018510	Suppression	Passive	3BNC117	30	8 viremic individuals	One dose	Some	Viral load was reduced by mean of 1.5 log_10_ copies/ml (range of 0.8–2.5), and significant for 28 days	[57]
NCT02446847	Maintenance	Passive	3BNC117	30	13 suppressed individuals	2 doses 3 weeks apart, or 4 doses 2 weeks apart; ART discontinued 2 days after first dose	All	2 infusions delayed rebound by mean of 6.7 weeks (range of 5–9) after ATI, and 4 infusions by mean of 9.9 weeks (range 3–19)	[58]
NCT01950325 (VRC 601)	Suppression	Passive	VRC01	40	8 viremic individuals	One dose	None	Amongst responders (6/8), viral load was reduced by 1.1–1.8 log_10_ copies/ml, and significant for 21 days	[55]
NCT02463227 (ACTG A5340)	Maintenance	Passive	VRC01	40	14 suppressed individuals	Dose 1 week before and 2 and 5 weeks after ART discontinuation	None	Rebound was delayed by 4 weeks (IQR 3–5) after ATI	[56]
NCT02471326 (NIH 15-I-0140)	Maintenance	Passive	VRC01	40	10 suppressed individuals	Dose 3 days before, 2 weeks after, and each subsequent month after ART discontinuation	None	Rebound was delayed by 5.6 weeks (IQR 4.1–5.6) after ATI	[56]
NCT02511990	Suppression	Passive	10-1074	30	13 viremic individuals	One dose	None	Amongst responders (11/13), viral load was reduced by mean of 1.5 log_10_ copies/ml (range 0.9–2.1), and significant for 27 days	[59]

The first clinical trials in HIV-1 infected individuals examined CD4 binding site bNAbs. In a phase I trial, Caskey et al. [57] administered a single infusion of 3BNC117 to eight HIV-1 infected, viremic individuals, which significantly reduced mean viremia from baseline for 4 weeks by up to 1.5 log_10_ copies/ml. A subsequent 3BNC117 ATI phase IIa trial gave multiple infusions in 13 HIV-1 infected individuals [58]. Individuals were pre-screened for PMBC viral outgrowth cultures with 3BNC117 sensitivity (IC_50_ ≤ 2.0 μg/ml). Viral rebound was significantly delayed by a mean of 6.7 weeks in individuals with 2 infusions, or by a mean of 9.9 weeks in individuals with 4 infusions, compared to historical controls of 2.6 weeks. 3BNC117 levels at viral rebound ranged from 6 to 168 μg/ml, and these values correlated with the IC_80_ of recrudescent viruses.

Two findings from these 3BNC117 trials suggested that 3BNC117 imposed a high barrier to viral escape. First, in a majority of participants of the second ATI trial (8/13), the recrudescent viruses were at least threefold more resistant by IC_80_. In 5 out of these 8, the rebounded virus appeared to have emerged from a single resistant provirus. In contrast, virus rebound after standard analytical treatment interruption without additional treatment is typically polyclonal, as multiple latent viruses are reactivated [61]. The restriction of recrudescent viruses suggests that 3BNC117 was preventing the rise of most latent clones. The virus that rebounded would have pre-existed at low frequencies such as not to have decreased bulk pre-infusion virus neutralizability. The first trial similarly found that recrudescent viruses with a reduction in 3BNC117 sensitivity tended to cluster in low-diversity lineages. Second, in the ATI trial, of the four individuals who were suppressed until antibody concentration fell below 20 μg/ml, three appeared to not have gained 3BNC117 resistance. Thus, a resistant mutant failed to emerge in the presence of the antibody, demonstrating antibody potency.

Three phase I trials of VRC01 passive infusion found that VRC01 could similarly suppress HIV-1, although suppression seemed to be less than that of 3BNC117. In Lynch et al. [55], in the 8 viremic patients given the highest dose, mean viral load was lower than baseline for 3 weeks. Individuals appeared to follow one of three patterns. Two individuals with mostly pre-existing resistant viruses did not respond, and two individuals with baseline viral loads less than 1000 copies/ml were briefly undetectable and then remained below baseline for at least 6 weeks. The last four individuals had sensitive viruses with a 14 to 59-fold reduction in viral load, but their virus started rebounding after 10 days. Except in the first two individuals with already completely resistant viruses, rebounded viruses had decreased sensitivity to VRC01 after infusion. These rebounded viruses were polyclonal except in one individual, where a pre-existing resistant minor lineage expanded to dominate the population.

In the two other VRC01 ATI trials, viral rebound was delayed by 4 or 5.6 weeks [56]. In a majority of individuals, the rebounded virus was polyclonal. Individuals with detected pre-existing resistant viruses had earlier viral rebounds, and individuals with pre-existing resistance throughout their viral diversity were more likely to have a polyclonal rebound. VRC01 resistance increased in most participants after infusion.

The 10-1074 trial tested a bNAb with an epitope outside of the CD4 binding site [59]. In this trial, 11 out of the 13 individuals who received the highest dose responded with a mean decrease of 1.5 log_10_ copies/ml, and the decrease was significant for almost four weeks. The two other individuals harbored pre-existing resistance and did not respond. Mean serum concentration was 77 μg/ml at rebound.

Across all VRC01 and 3BNC117 trials, mutations were observed occurring in or near the V5 loop, the D loop, and the CD4 binding site, epitopes common to CD4 binding site antibodies. In contrast, escape mutations in the 10-1074 trial were concentrated to the well-defined potential N-linked glycosylation N332 sequon (PNGS) and a ^324^G(D/N)IR^327^ motif. Resistance to 10-1074 was highly polyclonal in individual patients, suggesting that there were multiple ways the virus could escape antibody neutralization without greatly sacrificing viral infectivity and replicative fitness. Interestingly, the authors also found that baseline codon composition at these sites influenced the observed escaped mutations. Specifically, one individual, who in pre-treatment harbored a TCT serine codon instead of the more common AGT serine codon at the S334 PNGS, exhibited single point mutations at that codon to a different set of amino acids than other individuals after 10-1074 infusion. Lastly, in 5 out of 6 individuals sequenced after 10-1074 was no longer detectable, the N332 sequon and ^324^G(D/N)IR^327^ motif re-emerged, suggesting these escape mutations have sufficient in vivo fitness cost to necessitate reversion when antibody levels diminished.

Overall, these bNAbs are at least transiently efficacious in the therapeutic setting of suppressing viremia or preventing the emergence of latent viruses. In the case of 3BNC117 and VRC01, in patients where rebound virus emerged with reduced sensitivity to the infused bNAb, the rebound strains were often nearly identical and clustered together in low-diversity lineages in phylogenetic trees, distinct from the pre-existing quasispecies. This suggests that the bNAb bottlenecked the rebound virus—one or only a few strains escaped antibody pressure and subsequently expanded. These strains may have either been pre-existing at low-frequencies, or they represent chance emergence of resistant mutants during antibody therapy. In contrast, the rebound virus from 10-1074 was consistently polyclonal, suggesting that the barrier to escape may be lower for this bNAb. Whether this is because the 10-1074 epitope faces less selection pressure to remain as conserved as the CD4 binding site is unclear.

These clinical trials also found evidence that bNAbs have additional advantages over ARTs against HIV-1. In particular, 3BNC117 was found to have in vivo functionality beyond neutralization. 3BNC117 improved the anti-HIV-1 neutralization activity of autologous antibody responses and also increased clearance of infected cells through Fcɣ receptor engagement [62, 63]. Further investigation of how these antibodies may engage additional innate immune functions such as antibody-dependent cellular cytotoxicity and phagocytosis in vivo is needed [64, 65]. This is particularly important in the context of HIV-1 cure as antibodies may target cells within the latent viral reservoir that have been reactivated to produce virus [66].

In contrast to clinical trials for therapy, clinical trials for prevention are harder to conduct, as many more patients need to be repeatedly reinfused to detect treatment significance. The ongoing Antibody Mediated Prevention (AMP) study (HVTN 703/HPTN 081, https://clinicaltrials.gov/ct2/show/NCT02568215 and HVTN 704/HPTN 085, https://clinicaltrials.gov/ct2/show/NCT02716675) seeks to passively infuse VRC01 into thousands of trial participants every other month over the course of 10 infusions (Fig. 1a). Results from these trials are eagerly anticipated, as they are poised to be the first to show that bNAbs can actually prevent HIV infection in humans. Regardless of the outcomes of these trials, implementing continuous passive infusion globally, which requires repeated hospital visits from patients and cold chain transport, is infeasible. Steady-state antibody concentrations from passive transfer may also decline below prophylactic levels if the infusion schedule is delayed. Thus, vectored antibody delivery represents an attractive alternative for sustained bNAb production as a means of prevention. Sustained antibody levels, potentially achievable with gene transfer, may also result in long-term viral suppression, as suggested in the few 3BNC117 patients in whom viral rebound occurred only after antibody concentration greatly diminished and without increased resistance.

### Efficacy of vectored delivery of bNAbs in animal models

Gene delivery of bNAbs may result in the sustained, systemic expression of such antibodies with as little as one rAAV intramuscular injection, in contrast to passive transfer (Fig. 1). In this approach, antibodies are endogenously produced in muscle cells, targeted for export with secretion peptides, and passively circulated around the body. Table 2 summarizes the studies of vectored delivery of bNAbs covered in this review, and Table 3 lists considerations in using different animal models.

**Table 2:** Evaluation of vectored delivery of bNAbs against HIV-1 in animal models

Experimental design	Model	Delivery	Antibody	Highest rAAV genome copies given	Challenge virus	Challenge route	Week of 1st challenge post vectored delivery	[Antibody] in serum at challenge	Viremia in experimental arm	Viremia in control arm	References
Expression	Rag1 KO mice	rAAV2	b12	5 × 10^11^	HIV-1 IIIB	N/A (in vitro neutralization with serum)	20	2–6 μg/ml	1/6 not neutralize	No control	[67]
Protection	Macaques	rAAV1	4L6, 5L7, or N4 immunoadhesins	2 × 10^13^	SIVmac316	Intravenous	4	0–190 μg/ml (1/9 developed ADA)	3/9 after 1 challenge	6/6 after 1 challenge	[68]
Protection	HuPMBC-NSG mice	rAAV8	b12 or VRC01	1 × 10^11^	HIV NL4-3	Intravenous	4	198–313 μg/ml	0/8 after 1 challenge	8/8 after 1 challenge	[73]
Protection	BLT mice	rAAV8	VRC01	1 × 10^11^	HIV JR-CSF	Intravaginal	4	45–151 μg/ml	2/10 after 15 challenges	9/9, after mean 4.25 challenges	[77]
Protection	BLT mice	rAAV8	VRC07-G54W	1 × 10^11^	HIV REJO.c	Intravaginal	4	56–118 μg/ml	0/13 after 21 challenges	12/12, after mean 7.45 challenges	[77]
Maintenance	Hu-CD34-NSG mice	rAAV2	10-1074	2.5 × 10^11^	HIV YU-2-NL4-3	N/A	N/A	~ 200 μg/ml	1/7 rebounded	No control	[80]
Protection	Macaques	rAAV1	IgG1 versions of 4L6 or 5L7	1.6 × 10^13^	SIVmac239	Intravenous	14 or 44	0–270 μg/ml (9/12 developed ADA)	11/12 after 6 challenges	6/6 after 6 challenges	[106]
Protection	Macaques, with cyclosporine administration (CsA) for 4 weeks	rAAV8	Simianized VRC07	1 × 10^13^	SHIV-BaLP4	Intrarectal	5.5	0–39 μg/ml (1/6 developed ADA during CsA, 2/5 after CsA)	2/6 after 1 challenge	5/5 after 1 challenge	[107]
Protection	Macaques	rAAV1/2	Rhesus eCD4-Ig with rhesus tyrosine-protein sulfotransferase 2	2.5 × 10^13^	SHIV-AD8	Intravenous	8	17–77 μg/ml	0/4 after 6 challenges	4/4 after 6 challenges	[108]

**Table 3:** Considerations in model choice for evaluating vectored delivery of bNAbs against HIV-1

Characteristics	Hu-PBMC-NSG mice	Hu-CD34-NSG mice	Bone marrow-liver-thymus (BLT) mice	Macaques
Construction	Injection of expanded human PBMCs into NSG mice	Injection of fetal hu-CD34+ hematopoietic stem cells into newborn irradiated NSG mice	Injection of fetal hu-CD34+ hematopoietic stem cells into newborn irradiated NSG mice, along with surgical implantation of autologous thymus and liver tissue	Not needed
Antibody immunogenicity	(+) No strong anti-drug antibody response, permitting sustained antibody expression	(+) No strong anti-drug antibody response, permitting sustained antibody expression	(+) No strong anti-drug antibody response, permitting sustained antibody expression	(−) Strong anti-drug antibody responses, which is not fully resolved with antibody simianization
Immune functionality	(−) Activated human T-cell engraftment, but largely lacking other lineages, no hematopoietic regenerative source, no HLA-restriction, and no primary immune response	(-) Multi-lineage hematopoiesis with functional human T cell compartment that is mouse H2-restricted, but inconsistent humoral and Fc receptor effector responses	(+/−) Multi-lineage hematopoiesis with functional human T cell compartment that is HLA-restricted, but inconsistent humoral and Fc receptor effector responses	(+/−) Functional immune system, but is simian
Model longevity	(−) Up to 8 weeks due to GvHD	(+) Up to a year	(+) Up to a year	(+) Many years
HIV replication	(+) Supports HIV replication	(+) Supports HIV replication	(+) Supports HIV replication	(−) Does not support HIV replication (have to use either SIV or SHIV)
Challenge routes	(−) Intravenous only	(−) Intravenous only	(+) Intravenous, Intravaginal, Intrarectal	(+) Intravenous, Intravaginal, Intrarectal
Genetic homogeneity	(+) Genetic homogeneity within cohort with same graft, increasing reproducibility	(+) Genetic homogeneity within cohort with same graft, increasing reproducibility	(+) Genetic homogeneity within cohort with same graft, increasing reproducibility	(+/−) Genetic diversity, which can complicate analysis with small numbers
Similarity to human physiology and size	(−) Less	(−) Less	(−) Less	(+) More
Costs	(+) Lowest cost	(+) Low cost	(+/−) Moderate cost, but requires surgery	(−) High cost

Lewis et al. [67] first demonstrated sustained expression of a bNAb in mice in 2002. They delivered b12, a CD4 binding site bNAb, using an rAAV2 vector encoding both CMV and EF1-α promoters separately expressing the heavy and light chain genes. Injection into Rag1-immunodeficient mice resulted in peak serum levels of 4–9 μg/ml after 12 weeks, and the extracted serum was biologically active when measured by in vitro neutralization assays against HIV-1.

Johnson et al. [68] in 2009 subsequently delivered anti-SIV immunoadhesins via AAV in macaques. These immunoadhesins were based on anti-SIV Fabs obtained from PCR amplification of bone marrow cells of SIV-infected macaques and selected using phage display [69]. The variable light and heavy chains of these Fabs were joined by a linker to make a single chain variable fragment (scFv), which was then fused to a rhesus IgG2 Fc fragment. The authors constructed two such immunoadhesins, 4L6 and 5L7, as well as N4 that contained domains 1 and 2 of rhesus CD4. rAAVs encoding each of these constructs were intramuscularly injected into three macaques using an AAV1 capsid. N4 was constructed as a single-stranded genome, and 4L6 and 5L7 as self-complementary genomes, where two halves of an inverted repeat genome fold into a double-stranded DNA upon transduction, thereby bypassing the rate-limiting second-strand synthesis step. After 4 weeks, 4L6 or 5L7 immunoadhesin levels were 40–190 μg/ml, except for one macaque, in which 5L7 expression was eliminated due to the development of anti-5L7 antibodies. N4 levels were lower at 3–10 μg/ml. When these macaques were challenged intravenously with SIVmac316 a month after transduction, six out of nine animals were protected, but three were infected. Upon investigation, these three infected macaques had developed endogenous immunogenic responses against the immunoadhesins prior to challenge, thereby limiting the effectiveness of prevention.

In 2005, Fang et al. [70] first demonstrated the long-lasting delivery of full-length antibodies at therapeutic levels using single-stranded rAAV vectors. They achieved this by expressing a single open reading frame encoding the antibody heavy and light chains, linked by a 24-amino acid 2A self-processing sequence derived from picornavirus. Separation of these chains occurs between the last two residues of the 2A sequence through a ribosomal skip mechanism which prevents the formation of the peptide bond during translation [71]. A 4-amino acid furin cleavage sequence was added after the heavy chain and before the 2A sequence, which resulted in the removal of the residual 2A peptide in the Golgi. The single 2A amino acid at the N-terminus of the light chain is located prior to the signal peptide, and thus was not present in the mature antibody. Using this system, the authors demonstrated that injection of an rAAV8 vector carrying a VEGFR2-neutralizing antibody gene into mice resulted in > 1 mg/ml expression of the antibody for over 4 months with in vivo therapeutic efficacy. In a follow-up study, Fang et al. [72] optimized the furin cleavage site to achieve more complete and uniform cleavage.

Our lab used these developments to demonstrate that full-length bNAbs identical to those found in humans could be continuously produced at therapeutic levels via AAV gene transfer and that such vectored immunoprophylaxis (VIP) can prevent intravenous HIV transmission [73]. In addition to using codon-optimized 2A and furin sequences in the expression cassette, we also developed a muscle-optimized promoter (CASI), made from combining a CMV enhancer, a chicken β-actin promoter, and a ubiquitin enhancer embedded within a synthetic intron. A woodchuck hepatitis virus posttranscriptional regulatory element (WPRE) was included downstream of the antibody transgene to increase expression. For the vector, rAAV8 was used as it efficiently transduces non-dividing, post-mitotic muscle tissues, which have limited turnover, and human seroprevalence against AAV8 is lower than against AAV1 or AAV2 [74]. In addition, unlike AAV2, AAV8 does not activate capsid-specific T cells, due to lack of heparin binding which likely leads to uptake by dendritic cells [75], and may induce immune tolerance [10, 76]. In a first experiment, rAAV8-b12 was transduced into huPMBC-NSG humanized mice. Antibody levels were sustained at levels greater than 100 μg/ml a month after transduction. Transduced mice were fully protected from a challenge dose of NL4-3 HIV-1 that was 100-fold higher than needed to infect 7 out of 8 control mice. In a second dose-response experiment, the minimum amounts of rAAV-b12 or rAAV-VRC01 to fully protect mice from NL4-3 HIV-1 infection were found to be 1.25 × 10^10^ genome copies in both cases, corresponding to a mean concentration of 34 μg/ml for b12 and 8 μg/ml for VRC01.

Subsequently, our lab demonstrated that VIP can also protect humanized mice from low-dose repetitive intravaginal challenge [77]. To better model mucosal transmission of HIV-1, we used a more advanced bone marrow-liver-thymus (BLT) humanized mouse model, as described later in this review. In a first experiment, humanized mice transduced with rAAV8-VRC01 were challenged weekly with JR-CSF, a clade B, R5-tropic virus, starting a month after transduction. Upon transduction, VRC01 was detected at 100 μg/ml in the serum and a minimum of 100 ng/ml in the cervicovaginal lavage fluid, which represented an underestimate of the actual mucosal concentration as antibody was diluted by the vaginal wash procedure. Control mice were infected after a mean of 4.25 exposures, whereas only two of ten transduced mice were infected after 13 and 15 exposures. In a second experiment, VRC07-G54W was delivered via rAAV8 to humanized mice a month before beginning weekly challenges with a transmitted founder clade B, R5-tropic virus, REJO.c. VRC07 was created by pairing the original VRC01 light chain with a newly discovered heavy chain from the VRC01 patient [78], and the G54W mutation increased the antibody potency via mimicry of Phe43 in CD4 [79]. In this experiment, control mice were infected after a mean of 7.45 exposures, whereas none of the mice given VRC07-G54W antibody were infected after 21 exposures. These works demonstrated the protective effect of bNAbs delivered via rAAV against intravaginal HIV-1 challenge in a humanized mouse model.

Other labs have shown that rAAV-delivered bNAbs can also be used for therapeutic purposes. Horwitz et al. [80] in 2013 demonstrated that suppression of HIV-1 can be maintained with rAAV gene transfer of bNAbs in the NSG-CD34+ humanized mouse model. Because they found that ART interfered with AAV transduction, they first suppressed the virus with ART, then passively infused a bNAb while withdrawing ART, and subsequently maintained suppression with rAAV delivery of the same bNAb. They found that rAAV2-10-1074 sustained antibody concentrations of approximately 200 μg/ml and maintained suppression of YU-2-NL4-3 HIV-1 in 6 out of 7 mice. Future work could explore the use of other ART combinations to eliminate the need for a passive infusion bridge.

These studies demonstrate that rAAV delivery can sustain expression of anti-HIV-1 bNAbs in humanized mouse models. Similar VIP approaches have also been shown to protect mouse models against other infectious diseases, such as influenza [81], malaria [82], HCV [83], and Ebola [84].

### Evaluating vectored delivery in humanized mouse models

The natural immune response to HIV-1 is reflected to different degrees amongst the various humanized mouse models. The simplest mouse model infuses adult human T-cells derived from PBMCs into immunodeficient mice [85]. These mice support HIV-1 infection and ongoing viral replication, but the graft quickly depletes after several weeks as there is no regenerative source of T-cells. Furthermore, these mice develop graft-versus-host disease (GvHD) within 6–8 weeks as the graft is not tolerant of the foreign environment [73]. Another model involves the transplantation of human CD34+ stem cells into newborn immunodeficient mice [80, 86, 87]. This allows for the development of a regenerating T-cell compartment and improved longevity as a consequence of T-cell tolerance, which may be a consequence of human T-cell progenitors being educated in mouse thymus tissue. However, the lack of human thymus results in an immune system that is unable to recognize peptides presented in the context of human HLA molecules and thus a largely incompetent adaptive immune response to infection. The most complete humanized mouse model is the bone marrow-liver-thymus (BLT) mouse model, in which newborn immunodeficient mice are surgically implanted with tissue fragments from human fetal liver and thymus, followed by intravenous injection of purified autologous fetal human CD34+ hematopoietic stem cells derived from the remainder of unimplanted liver [88–90]. The T cell compartment of the BLT mice reconstitutes over several months and T cells mature in the transplanted human thymus and are largely tolerant to mouse antigens. The BLT mouse can model multiple aspects of HIV-1 infection, such as prevention, viral evolution in response to T-cell pressure, mucosal transmission, CTL responses, and viral latency [77, 91–94].

Humanized mouse models have the benefit of genetic homogeneity when engrafted with tissue from the same donor. Isogenic cohorts can be as large as tens to over one hundred and fifty mice, allowing for the observation of chance behaviors in response to HIV-1 infection. These humanized mouse models are also significantly less expensive than macaque models. Importantly, natural human bNAbs with specificity against HIV can be tested in humanized mice without eliciting strong ADA responses that confound experiments in other systems.

However, the existing BLT mouse model does not yet faithfully replicate all aspects of a fully-functional human immune system. Our lab and others have observed inconsistent humoral responses against viral proteins in BLT mice during HIV-1 infection. This could be due to previous observations of disorganized germinal centers, and defects in antigen presentation. In addition, the BLT model does not fully capture the pharmacokinetics and pharmacodynamics of human bNAbs given the murine origin of the neonatal Fc receptor (FcRn) recycling receptors [95]. Fc-mediated behaviors beyond neutralization, such as antibody-dependent cell-mediated cytotoxicity (ADCC) may not be well supported in this model due to a paucity of natural killer cells. Furthermore, complement-dependent cytotoxicity (CDC) is not supported in this model due to genetic defects in the underlying NSG mouse strain complement cascade [96].

### Evaluating vectored delivery of bNAbs in macaques

Evaluating the effectiveness of rAAV gene transfer of anti-HIV-1 bNAbs in nonhuman primates has proven to be challenging, due to a lack of naturally-existing, effective antibodies against SIV and the propensity of macaques to develop strong immunogenic responses against human bNAbs. However, macaques are better models of human physiology, have fully functional immune systems that are analogous to that of humans, and are of more comparable size. Importantly, HIV-1 does not replicate in macaques and thus a closely related virus, SIVmac, is used to model HIV-1 infection and the corresponding AIDS-like symptoms that develop [97]. However, while functionally similar, SIV and HIV-1 share only about 50% sequence homology, and SIV encodes an additional accessory protein (Vpx), not found in HIV-1, that induces degradation of host restriction factor SAMHD1 [98, 99].

Given that human bNAbs do not recognize SIVmac, a chimeric virus (SHIV) is often used for antibody-mediated protection experiments in which the SIVmac envelope is replaced with an HIV-1 envelope [100]. However, this chimeric virus is not fully adapted and may be of lower fitness than natural SIV strains, as unlike HIV-1 in humans or in BLT humanized mice, some SHIV strains are occasionally controlled in untreated macaques [101, 102]. Interestingly, not all HIV-1 envelopes can be made into functional SHIV, although substantial progress has been made in doing so [103]. In addition, unlike mice, macaques are not inbred, and genetic diversity in a cohort may lead to disparate immunological responses to infection that can complicate analysis of divergent behavior within a group. Most importantly, as reviewed in this section, human bNAbs appear to elicit significant immunogenic responses in macaques [104, 105], and these responses are not seen when passively transferring bNAbs in humans.

Fuchs et al. [106] in 2015 constructed full-length rhesus IgG1 counterparts of the 4L6 or 5L7 immunoadhesins used in Johnson et al. [68] and delivered them via rAAV1 to macaques. Although antibody concentrations reached 20–300 μg/ml, endogenous ADA responses were detected within a month in three of the six macaques given 5L7, and in all six macaques given 4L6. These ADA responses limited continual expression of the antibody, as antibody concentrations fell below 10 μg/ml in macaques that developed ADA responses. The authors then repeatedly challenged the macaques with SIVmac239, a strain more difficult to neutralize than SIVmac319 used by Johnson et al. Although neither gene transfer appeared to be more effective at preventing infection than the negative control, 5L7 delayed time to peak viral load, and lowered peak and set viral loads. It was later found that the variable regions of these antibodies were immunogenic, which contributed to their xenogeneic elimination [105].

Other works have sought to simianize bNAbs to reduce cross-species immunogenicity with mixed results. Saunders et al. [104] in 2015 found that a single infusion of human VRC01 resulted in detectable anti-VRC01 plasma IgG eight weeks after infusion, but not for simianized VRC01. They subsequently infused simianized simVRC01 or simVRC01-LS four times over 8 months into eight macaques, and found that the bNAb could persist for more than 2 or 3.5 months, respectively, after the last infusion. However, in two of the eight macaques, ADA response was nonetheless detected against the simianized antibody. The six other macaques were intrarectally challenged with SHIV-BaLP4 2 months after last the passive infusion, of which five were protected.

In a parallel study, Saunders et al. [107] evaluated whether administering immunosuppressant cyclosporine before rAAV injection reduced immunogenicity against bNAbs. The authors first delivered simianized VRC07 via rAAV8 to macaques. Serum concentrations peaked at 2.5–7.7 μg/ml at weeks 2–4, and substantial ADA response was detected. The same constructs given at a lower dose to immunodeficient mice resulted in levels greater than 100 μg/ml. In a second study, six macaques were given cyclosporine starting 9 days prior to, and until 4 weeks after, rAAV8-simVRC07 administration. Peak mean concentration of simVRC07 was 38 μg/ml. Three of six macaques retained simVRC07 expression for 16 weeks without developing ADA, whereas the others exhibited ADA, including one that completely eliminated simVRC07 expression. Macaques were intrarectally challenged with SHIV-BaLP4 5.5 weeks after transduction, and the two of the six macaques with the lowest simVRC07 concentrations became infected. Overall, ADA response was inversely correlated with simVRC07 concentration, and transient immunosuppression did increase transgene expression.

Since bNAbs, even simianized, were still immunogenic after long-term expression in macaques, Gardner et al. [108] in 2015 took an alternative approach and delivered rh-eCD4-Ig, which is rhesus CD4-Ig fused at its carboxyl terminus to a 15-amino acid CCR5-mimetic sulfopeptide, in an rAAV1/2 vector into macaques. Rhesus tyrosine-protein sulfotransferase 2 was also co-administered in a second rAAV at a 1:4 ratio to increase rh-CD4-Ig sulfation, as this is required for its neutralizing activity. rh-eCD4-Ig was expressed at 17-77 μg/ml for more than 40 weeks and protected four macaques from four increasingly stringent challenges with SHIV-AD8. Less anti-transgene response was detected against rh-eCD4-Ig than against simianized 3BNC117, NIH45-46, 10-1074, PGT121, and no antibody response against the sulfopeptide was detected.

In summary, there are substantial challenges associated with evaluating the long-term expression of bNAbs in nonhuman primates, through both repeated passive infusions and rAAV gene delivery. Although simianization, in which human variable regions are engrafted onto a rhesus antibody, reduces bNAb immunogenicity in passive transfer studies, it does not fully eliminate ADA responses as those variable regions are descended from the human germline and not from their simian counterparts. Thus, it may be inherently difficult to evaluate the anti-HIV-1 efficacy of sustained human bNAb expression in macaques with confounding xenogenic responses. Immunosuppressants such as cyclosporine reduce but do not completely eliminate the immunogenicity of bNAbs [107]. Future studies aiming to deliver fully simian antibodies against SIV or HIV, cloned in a manner analogous to methods used to isolate human bNAbs, may more accurately predict the potential for translation of this approach in humans.

### Vectored delivery of bNAbs in clinical trials

There are currently only two bNAb AAV gene transfer studies. The first trial (IAVI A003/CHOP HVDDT 001, https://clinicaltrials.gov/ct2/show/NCT01937455) is a phase I trial in 21 healthy males without HIV-1 or HIV-2 infection and uses an rAAV1 vector encoding PG9 heavy and light chain under two separate promoters. Either 4 × 10^12^, 4 × 10^13^, 8 × 10^13^, or 1.2 × 10^14^ viral genomes regardless of weight or placebo were given in a single intramuscular administration to participants without evidence of pre-existing anti-AAV1 antibodies. These participants were then followed for a year, with the option of enrolling into a follow-up study. This trial was completed in February 2018, but results have not yet been reported in the literature. The second trial (VRC 603, https://clinicaltrials.gov/ct2/show/NCT03374202) is a phase I trial in an estimated 25 adults living with suppressed HIV-1 infection and uses an rAAV8 vector encoding a CASI-promoter driven VRC07 transgene in a nearly identical configuration to those used in our previously published studies [73, 77]. Participants in VRC603 must have controlled viremia, have been on stable ART for at least 3 months, and not have evidence of pre-existing anti-AAV8 antibodies. Either 5 × 10^10^, 5 × 10^11^, or 2.5 × 10^12^ viral genomes per kg will be given in a single intramuscular administration to the upper arm or thigh, with a study goal of achieving 50 μg/ml 4 weeks post-injection and a set point of 5 μg/ml 12 weeks post-injection. Participants will be followed intensely for a year, and then every 6 months for another 4 years. This trial has an estimated primary completion date of March 2019. Longer-term follow up of patients in these trials, past these primary study completion dates, is desirable to evaluate the duration of sustained bNAb expression and the potential immunogenic responses from chronic bNAb exposure.

### Selection of bNAbs for vectored delivery

Since the characterization of PG9 in 2009 [109], over 90 bNAbs have been described, exhibiting a wide range of breadth and potency as determined by neutralization assays on large global panels of HIV-1 isolates [42, 110]. Given that most infections are initiated by a single transmitted founder virus [111, 112], low in vivo bNAb concentrations that reduce the probability of the establishment of infection may be sufficient to provide a benefit in the context of prevention. As more potent antibodies are discovered, the prophylactic dose of bNAb necessary to yield protective concentrations is likely to be reduced.

In contrast, suppressing actively replicating virus with bNAb is more challenging given the millions to billions of virions that must be neutralized. In addition, instead of a single viral genotype, the bNAb is faced with neutralizing a quasispecies of closely related HIV-1 strains [113]. Since the virus mutates as it replicates, the quasispecies may harbor a variant that evades antibody neutralization, thereby allowing for escape. Such variants that escape bNAb pressure may face a replicative fitness penalty, particularly if conserved sites such as those involved in interacting with CD4 are mutated. It may be useful to consider the fitness costs of escaping each bNAb, and use antibodies that impose a high escape cost when optimizing bNAb delivery, particularly in a therapeutic setting [114, 115].

Another feature specific to rAAV delivery is that bNAb expression may take several weeks to achieve steady state levels [73, 77], as second strand synthesis of the rAAV genome is necessary for expression to occur [26, 27]. From the viral perspective, this steadily rising concentration of antibody represents a gradually increasing selective force which may more readily select for escape mutants. Use of a stronger promoter or of a more efficient AAV serotype may result in faster expression. Alternatively, co-adminstration of both an adenovirus vector and an AAV vector can result in immediate and sustained antibody expression, as previously shown for a monoclonal antibody against anthrax [116].

Similar to existing HAART regimens, which employ a combination of antiretroviral drugs to control HIV-1, the use of antibody combinations to suppress HIV-1 has been long proposed and may be necessary, particularly in the context of therapy [117, 118]. Klein et al. [119] repeatedly passively infused either a tri-mix or penta-mix of bNAbs into YU-2-NL4-3 HIV-1 infected humanized mice. The tri-mix (which neutralized > 98% of clades with IC_80_ of 0.121 μg/ml) led to complete suppression in 3 of 12 mice, and the penta-mix (which neutralized > 98% of clades with IC_80_ of 0.046 μg/ml) led to complete suppression in 11 of 13 mice. This and other work suggests that antibodies which bind to different epitopes and with very low IC_50_ across diverse HIV-1 strains should be chosen [119–121]. Bispecific monoclonal antibodies, where each arm of the antibody binds to a different epitope, may also have greater breath and potency than each constituent antibody alone or mixed [122, 123]. rAAV delivery of bispecifics may require two separate rAAVs injected into the same site, due to the carrying capacity of the vector, but this has not yet been reported in the literature. Another approach is to study viral mutants that arise after administration of a single bNAb and then design variants of that bNAb that neutralize those mutants. In particular, Diskin et al. [124] rationally designed NIH45-46 variants by increasing the buried surface area of the antibody with escape variants and avoiding steric clashes. However, when NIH45-46 and its variants were passively infused as a combination into infected humanized mice, mutants escaped in a previously unseen path by shifting an N-linked glycosylation site by three residues, highlighting the magnitude of the challenge of designing antibody combinations to suppress actively evolving viremia.

## Conclusion

Phase I and IIa trials of passive transfer of bNAbs have thus far demonstrated the safety of bNAbs in humans and shown that bNAbs can both transiently lower viral loads and delay viral rebound. In some patients, viral rebound happened only after the bNAb concentration fell to low concentrations and occurred without escape mutations, suggesting that continuous bNAb expression might result in sustained suppression. Given the difficulty of eliciting bNAbs through sequential vaccination and the complexity of life-long passive transfer of bNAbs, using a single intramuscular administration of rAAV to attain continuous, systemic, long-term expression of bNAbs is an exciting possibility.

rAAVs have a favorable safety profile and can stably express transgenes in humans for many years. However, pre-existing immunity in individuals against AAV due to natural exposure may limit successful vectored delivery. To avoid this, immunosuppressants may be temporarily administered, or novel AAV capsids with little cross-reactivity to circulating AAVs may be used [125]. In humanized mouse models, vectored delivery resulted in the long-term expression of bNAbs, protected against intravenous and intravaginal HIV-1 challenges, and maintained suppression of previously ART-suppressed HIV-1. However, pre-existing and emergent viral resistance to bNAbs may limit their effectiveness in patients. Whereas bNAbs may be able to neutralize a few slightly resistant virions in the context of prevention, using bNAbs to suppress replicating HIV-1 may require additional innovations to prevent the evolution and selection of viral mutants. Prevention studies in macaques with AAV-delivered simianized bNAbs elicited strong anti-bNAb responses, although it seems likely that the immunogenicity of the human antibody variable regions in macaques complicates this model. Most importantly, Phase I clinical trials of vectored delivery of bNAbs are currently underway and should provide critically important information to determine the feasibility of this approach. Irrespective of their outcome, we believe that whatever challenges may be encountered will ultimately be overcome and that vectored delivery of broadly neutralizing antibodies will become an important new approach towards ending the HIV-1 epidemic.

## Abbreviations

AAV: adeno-associated virus
ADA: anti-drug antibody
ART: antiretroviral therapy
ATI: analytical treatment interruption
BCR: B-cell receptor
BLT: bone marrow-liver-thymus
bNAb: broadly neutralizing antibody
HIV: human immunodeficiency virus
ITR: inverted terminal repeat
PNGS: potential N-linked glycosylation sequon
rAAV: recombinant adeno-associated virus
SIV: simian immunodeficiency virus
VIP: vectored immunoprophylaxis

## References

[1] UNAIDS. Global AIDS Update. Sep 2017.

[2] Hermonat PL, Muzyczka N (1984). Use of adeno-associated virus as a mammalian DNA cloning vector: transduction of neomycin resistance into mammalian tissue culture cells. Proc Natl Acad Sci USA.

[3] Casto BC, Hammon WM, Atchison RW (1965). Adenovirus-associated defective virus particles. Science.

[4] Lusby E, Fife KH, Berns KI (1980). Nucleotide sequence of the inverted terminal repetition in adeno-associated virus DNA. J Virol.

[5] Sonntag F, Schmidt K, Kleinschmidt JA (2010). A viral assembly factor promotes AAV2 capsid formation in the nucleolus. Proc Natl Acad Sci USA.

[6] Zincarelli C, Soltys S, Rengo G, Rabinowitz JE (2008). Analysis of AAV serotypes 1–9 mediated gene expression and tropism in mice after systemic injection. Mol Ther.

[7] Gao G, Alvira MR, Somanathan S, Lu Y, Vandenberghe LH, Rux JJ (2003). Adeno-associated viruses undergo substantial evolution in primates during natural infections. Proc Natl Acad Sci USA.

[8] Gao G, Vandenberghe LH, Alvira MR, Lu Y, Calcedo R, Zhou X (2004). Clades of adeno-associated viruses are widely disseminated in human tissues. J Virol.

[9] Zinn E, Pacouret S, Khaychuk V, Turunen HT, Carvalho LS, Andres-Mateos E (2015). In silico reconstruction of the viral evolutionary lineage yields a potent gene therapy vector. Cell Rep.

[10] Mays LE, Vandenberghe LH, Xiao R, Bell P, Nam H-J, Agbandje-McKenna M (2009). Adeno-associated virus capsid structure drives CD4-dependent CD8+ T cell response to vector encoded proteins. J Immunol.

[11] Mays LE, Wang L, Tenney R, Bell P, Nam H-J, Lin J (2013). Mapping the structural determinants responsible for enhanced T cell activation to the immunogenic adeno-associated virus capsid from isolate rhesus 32.33. J Virol.

[12] Kotterman MA, Schaffer DV (2014). Engineering adeno-associated viruses for clinical gene therapy. Nat Rev Genet.

[13] Berns KI, Parrish CR, Knipe DM, Howley PM, Cohen JI, Griffin DE, Lamb RA, Martin MA (2013). Parvoviridae. Fields virology.

[14] Kotin RM, Siniscalco M, Samulski RJ, Zhu XD, Hunter L, Laughlin CA (1990). Site-specific integration by adeno-associated virus. Proc Natl Acad Sci USA.

[15] Samulski RJ, Zhu X, Xiao X, Brook JD, Housman DE, Epstein N (1991). Targeted integration of adeno-associated virus (AAV) into human chromosome 19. EMBO J.

[16] Surosky RT, Urabe M, Godwin SG, McQuiston SA, Kurtzman GJ, Ozawa K (1997). Adeno-associated virus Rep proteins target DNA sequences to a unique locus in the human genome. J Virol.

[17] Schnepp BC, Chulay JD, Ye G-J, Flotte TR, Trapnell BC, Johnson PR (2016). Recombinant adeno-associated virus vector genomes take the form of long-lived, transcriptionally competent episomes in human muscle. Hum Gene Ther.

[18] Schnepp BC, Clark KR, Klemanski DL, Pacak CA, Johnson PR (2003). Genetic fate of recombinant adeno-associated virus vector genomes in muscle. J Virol.

[19] Nowrouzi A, Penaud-Budloo M, Kaeppel C, Appelt U, Le Guiner C, Moullier P (2012). Integration frequency and intermolecular recombination of rAAV vectors in non-human primate skeletal muscle and liver. Mol Ther.

[20] Herzog RW, Yang EY, Couto LB, Hagstrom JN, Elwell D, Fields PA (1999). Long-term correction of canine hemophilia B by gene transfer of blood coagulation factor IX mediated by adeno-associated viral vector. Nat Med.

[21] Xiao X, Li J, Samulski RJ (1996). Efficient long-term gene transfer into muscle tissue of immunocompetent mice by adeno-associated virus vector. J Virol.

[22] Rivera VM, Gao G-P, Grant RL, Schnell MA, Zoltick PW, Rozamus LW (2005). Long-term pharmacologically regulated expression of erythropoietin in primates following AAV-mediated gene transfer. Blood.

[23] Nathwani AC, Reiss UM, Tuddenham EGD, Rosales C, Chowdary P, McIntosh J (2014). Long-term safety and efficacy of factor IX gene therapy in hemophilia B. N Engl J Med.

[24] Buchlis G, Podsakoff GM, Radu A, Hawk SM, Flake AW, Mingozzi F (2012). Factor IX expression in skeletal muscle of a severe hemophilia B patient 10 years after AAV-mediated gene transfer. Blood.

[25] Dong JY, Fan PD, Frizzell RA (1996). Quantitative analysis of the packaging capacity of recombinant adeno-associated virus. Hum Gene Ther.

[26] Ferrari FK, Samulski T, Shenk T, Samulski RJ (1996). Second-strand synthesis is a rate-limiting step for efficient transduction by recombinant adeno-associated virus vectors. J Virol.

[27] Fisher KJ, Gao GP, Weitzman MD, DeMatteo R, Burda JF, Wilson JM (1996). Transduction with recombinant adeno-associated virus for gene therapy is limited by leading-strand synthesis. J Virol.

[28] Boutin S, Monteilhet V, Veron P, Leborgne C, Benveniste O, Montus MF (2010). Prevalence of serum IgG and neutralizing factors against adeno-associated virus (AAV) types 1, 2, 5, 6, 8, and 9 in the healthy population: implications for gene therapy using AAV vectors. Hum Gene Ther.

[29] Calcedo R, Vandenberghe LH, Gao G, Lin J, Wilson JM (2009). Worldwide epidemiology of neutralizing antibodies to adeno-associated viruses. J Infect Dis.

[30] Manno CS, Pierce GF, Arruda VR, Glader B, Ragni M, Rasko JJ (2006). Successful transduction of liver in hemophilia by AAV-Factor IX and limitations imposed by the host immune response. Nat Med.

[31] George LA, Sullivan SK, Giermasz A, Rasko JEJ, Samelson-Jones BJ, Ducore J (2017). Hemophilia B gene therapy with a high-specific-activity factor IX variant. N Engl J Med.

[32] Naso MF, Tomkowicz B, Perry WL, Strohl WR (2017). Adeno-associated virus (AAV) as a vector for gene therapy. BioDrugs.

[33] Maguire AM, Simonelli F, Pierce EA, Pugh EN, Mingozzi F, Bennicelli J (2008). Safety and efficacy of gene transfer for Leber’s congenital amaurosis. N Engl J Med.

[34] Bainbridge JWB, Smith AJ, Barker SS, Robbie S, Henderson R, Balaggan K (2008). Effect of gene therapy on visual function in Leber’s congenital amaurosis. N Engl J Med.

[35] Senior M (2017). After Glybera’s withdrawal, what’s next for gene therapy?. Nat Biotechnol.

[36] Spark’s gene therapy price tag: $850,000. Nat Biotechnol. 2018;36:122–2. 10.1038/nbt0218-122. 10.1038/nbt0218-122 29406495

[37] Hraber P, Seaman MS, Bailer RT, Mascola JR, Montefiori DC, Korber BT (2014). Prevalence of broadly neutralizing antibody responses during chronic HIV-1 infection. AIDS.

[38] Liao H-X, Lynch R, Zhou T, Gao F, Alam SM, Boyd SD (2013). Co-evolution of a broadly neutralizing HIV-1 antibody and founder virus. Nature.

[39] Zhu P, Liu J, Bess J, Chertova E, Lifson JD, Grisé H (2006). Distribution and three-dimensional structure of AIDS virus envelope spikes. Nature.

[40] Wei X, Decker JM, Wang S, Hui H, Kappes JC, Wu X (2003). Antibody neutralization and escape by HIV-1. Nature.

[41] Rusert P, Krarup A, Magnus C, Brandenberg OF, Weber J, Ehlert A-K (2011). Interaction of the gp120 V1V2 loop with a neighboring gp120 unit shields the HIV envelope trimer against cross-neutralizing antibodies. J Exp Med.

[42] Eroshkin AM, LeBlanc A, Weekes D, Post K, Li Z, Rajput A (2013). bNAber: database of broadly neutralizing HIV antibodies. Nucleic Acids Res.

[43] Wibmer CK, Moore PL, Morris L (2015). HIV broadly neutralizing antibody targets. Curr Opin HIV AIDS.

[44] Burton DR, Pyati J, Koduri R, Sharp SJ, Thornton GB, Parren PW (1994). Efficient neutralization of primary isolates of HIV-1 by a recombinant human monoclonal antibody. Science.

[45] Wu X, Yang Z-Y, Li Y, Hogerkorp C-M, Schief WR, Seaman MS (2010). Rational design of envelope identifies broadly neutralizing human monoclonal antibodies to HIV-1. Science.

[46] Zhou T, Georgiev I, Wu X, Yang Z-Y, Dai K, Finzi A (2010). Structural basis for broad and potent neutralization of HIV-1 by antibody VRC01. Science.

[47] Scheid JF, Mouquet H, Ueberheide B, Diskin R, Klein F, Oliveira TYK (2011). Sequence and structural convergence of broad and potent HIV antibodies that mimic CD4 binding. Science.

[48] Huang J, Kang BH, Ishida E, Zhou T, Griesman T, Sheng Z (2016). Identification of a CD4-binding-site antibody to HIV that evolved near-pan neutralization breadth. Immunity.

[49] Sajadi MM, Dashti A, Rikhtegaran Tehrani Z, Tolbert WD, Seaman MS, Ouyang X (2018). Identification of near-pan-neutralizing antibodies against HIV-1 by deconvolution of plasma humoral responses. Cell.

[50] Wang S, Mata-Fink J, Kriegsman B, Hanson M, Irvine DJ, Eisen HN (2015). Manipulating the selection forces during affinity maturation to generate cross-reactive HIV antibodies. Cell.

[51] Andrabi R, Bhiman JN, Burton DR (2018). Strategies for a multi-stage neutralizing antibody-based HIV vaccine. Curr Opin Immunol.

[52] Williams WB, Zhang J, Jiang C, Nicely NI, Fera D, Luo K (2017). Initiation of HIV neutralizing B cell lineages with sequential envelope immunizations. Nat Commun.

[53] Jardine JG, Kulp DW, Havenar-Daughton C, Sarkar A, Briney B, Sok D (2016). HIV-1 broadly neutralizing antibody precursor B cells revealed by germline-targeting immunogen. Science.

[54] Briney B, Sok D, Jardine JG, Kulp DW, Skog P, Menis S (2016). Tailored immunogens direct affinity maturation toward HIV neutralizing antibodies. Cell.

[55] Lynch RM, Boritz E, Tressler R, Coates EE, DeZure A, Bailer RT (2015). Virologic effects of broadly neutralizing antibody VRC01 administration during chronic HIV-1 infection. Sci Transl Med.

[56] Bar KJ, Sneller MC, Harrison LJ, Justement JS, Overton ET, Petrone ME (2016). Effect of HIV antibody VRC01 on viral rebound after treatment interruption. N Engl J Med.

[57] Caskey M, Klein F, Lorenzi JCC, Seaman MS, West AP, Buckley N (2015). Viraemia suppressed in HIV-1-infected humans by broadly neutralizing antibody 3BNC117. Nature.

[58] Scheid JF, Horwitz JA, Bar-On Y, Kreider EF, Lu C-L, Lorenzi JCC (2016). HIV-1 antibody 3BNC117 suppresses viral rebound in humans during treatment interruption. Nature.

[59] Caskey M, Schoofs T, Gruell H, Settler A, Karagounis T, Kreider EF (2017). Antibody 10-1074 suppresses viremia in HIV-1-infected individuals. Nat Med.

[60] Cohen YZ, Caskey M (2018). Broadly neutralizing antibodies for treatment and prevention of HIV-1 infection. Curr Opin HIV AIDS.

[61] Rothenberger MK, Keele BF, Wietgrefe SW, Fletcher CV, Beilman GJ, Chipman JG (2015). Large number of rebounding/founder HIV variants emerge from multifocal infection in lymphatic tissues after treatment interruption. Proc Natl Acad Sci USA.

[62] Schoofs T, Klein F, Braunschweig M, Kreider EF, Feldmann A, Nogueira L (2016). HIV-1 therapy with monoclonal antibody 3BNC117 elicits host immune responses against HIV-1. Science.

[63] Lu C-L, Murakowski DK, Bournazos S, Schoofs T, Sarkar D, Halper-Stromberg A (2016). Enhanced clearance of HIV-1-infected cells by broadly neutralizing antibodies against HIV-1 in vivo. Science.

[64] Burton DR, Mascola JR (2015). Antibody responses to envelope glycoproteins in HIV-1 infection. Nat Immunol.

[65] Bruel T, Guivel-Benhassine F, Amraoui S, Malbec M, Richard L, Bourdic K (2016). Elimination of HIV-1-infected cells by broadly neutralizing antibodies. Nat Commun.

[66] Halper-Stromberg A, Lu C-L, Klein F, Horwitz JA, Bournazos S, Nogueira L (2014). Broadly neutralizing antibodies and viral inducers decrease rebound from HIV-1 latent reservoirs in humanized mice. Cell.

[67] Lewis AD, Chen R, Montefiori DC, Johnson PR, Clark KR (2002). Generation of neutralizing activity against human immunodeficiency virus type 1 in serum by antibody gene transfer. J Virol.

[68] Johnson PR, Schnepp BC, Zhang J, Connell MJ, Greene SM, Yuste E (2009). Vector-mediated gene transfer engenders long-lived neutralizing activity and protection against SIV infection in monkeys. Nat Med.

[69] Johnson WE, Sanford H, Schwall L, Burton DR, Parren PWHI, Robinson JE (2003). Assorted mutations in the envelope gene of simian immunodeficiency virus lead to loss of neutralization resistance against antibodies representing a broad spectrum of specificities. J Virol.

[70] Fang J, Qian J-J, Yi S, Harding TC, Tu GH, VanRoey M (2005). Stable antibody expression at therapeutic levels using the 2A peptide. Nat Biotechnol.

[71] Donnelly ML, Luke G, Mehrotra A, Li X, Hughes LE, Gani D (2001). Analysis of the aphthovirus 2A/2B polyprotein ‘cleavage’ mechanism indicates not a proteolytic reaction, but a novel translational effect: a putative ribosomal ‘skip’. J Gen Virol.

[72] Fang J, Yi S, Simmons A, Tu GH, Nguyen M, Harding TC (2007). An antibody delivery system for regulated expression of therapeutic levels of monoclonal antibodies in vivo. Mol Ther.

[73] Balazs AB, Chen J, Hong CM, Rao DS, Yang L, Baltimore D (2011). Antibody-based protection against HIV infection by vectored immunoprophylaxis. Nature.

[74] Gao G-P, Alvira MR, Wang L, Calcedo R, Johnston J, Wilson JM (2002). Novel adeno-associated viruses from rhesus monkeys as vectors for human gene therapy. Proc Natl Acad Sci USA.

[75] Vandenberghe LH, Wang L, Somanathan S, Zhi Y, Figueredo J, Calcedo R (2006). Heparin binding directs activation of T cells against adeno-associated virus serotype 2 capsid. Nat Med.

[76] Mays LE, Wang L, Lin J, Bell P, Crawford A, Wherry EJ (2014). AAV8 induces tolerance in murine muscle as a result of poor APC transduction, T cell exhaustion, and minimal MHCI upregulation on target cells. Mol Ther.

[77] Balazs AB, Ouyang Y, Hong CM, Chen J, Nguyen SM, Rao DS (2014). Vectored immunoprophylaxis protects humanized mice from mucosal HIV transmission. Nat Med.

[78] Rudicell RS, Kwon YD, Ko S-Y, Pegu A, Louder MK, Georgiev IS (2014). Enhanced potency of a broadly neutralizing HIV-1 antibody in vitro improves protection against lentiviral infection in vivo. J Virol.

[79] Diskin R, Scheid JF, Marcovecchio PM, West AP, Klein F, Gao H (2011). Increasing the potency and breadth of an HIV antibody by using structure-based rational design. Science.

[80] Horwitz JA, Halper-Stromberg A, Mouquet H, Gitlin AD, Tretiakova A, Eisenreich TR (2013). HIV-1 suppression and durable control by combining single broadly neutralizing antibodies and antiretroviral drugs in humanized mice. Proc Natl Acad Sci USA.

[81] Balazs AB, Bloom JD, Hong CM, Rao DS, Baltimore D (2013). Broad protection against influenza infection by vectored immunoprophylaxis in mice. Nat Biotechnol.

[82] Deal C, Balazs AB, Espinosa DA, Zavala F, Baltimore D, Ketner G (2014). Vectored antibody gene delivery protects against Plasmodium falciparum sporozoite challenge in mice. Proc Natl Acad Sci USA.

[83] de Jong YP, Dorner M, Mommersteeg MC, Xiao JW, Balazs AB, Robbins JB (2014). Broadly neutralizing antibodies abrogate established hepatitis C virus infection. Sci Transl Med.

[84] van Lieshout LP, Soule G, Sorensen D, Frost KL, He S, Tierney K (2018). Intramuscular adeno-associated virus-mediated expression of monoclonal antibodies provides 100% protection against Ebola virus infection in mice. J Infect Dis.

[85] Mosier DE, Gulizia RJ, Baird SM, Wilson DB, Spector DH, Spector SA (1991). Human immunodeficiency virus infection of human-PBL-SCID mice. Science.

[86] Traggiai E, Chicha L, Mazzucchelli L, Bronz L, Piffaretti J-C, Lanzavecchia A (2004). Development of a human adaptive immune system in cord blood cell-transplanted mice. Science.

[87] Ishikawa F, Yasukawa M, Lyons B, Yoshida S, Miyamoto T, Yoshimoto G (2005). Development of functional human blood and immune systems in NOD/SCID/IL2 receptor γ chain^null^ mice. Blood.

[88] Lan P, Tonomura N, Shimizu A, Wang S, Yang Y-G (2006). Reconstitution of a functional human immune system in immunodeficient mice through combined human fetal thymus/liver and CD34+ cell transplantation. Blood.

[89] Melkus MW, Estes JD, Padgett-Thomas A, Gatlin J, Denton PW, Othieno FA (2006). Humanized mice mount specific adaptive and innate immune responses to EBV and TSST-1. Nat Med.

[90] Karpel ME, Boutwell CL, Allen TM (2015). BLT humanized mice as a small animal model of HIV infection. Curr Opin Virol.

[91] Dudek TE, No DC, Seung E, Vrbanac VD, Fadda L, Bhoumik P (2012). Rapid evolution of HIV-1 to functional CD8^+^ T cell responses in humanized BLT mice. Sci Transl Med.

[92] Denton PW, Olesen R, Choudhary SK, Archin NM, Wahl A, Swanson MD (2012). Generation of HIV latency in humanized BLT mice. J Virol.

[93] Marsden MD, Kovochich M, Suree N, Shimizu S, Mehta R, Cortado R (2012). HIV latency in the humanized BLT mouse. J Virol.

[94] Denton PW, Estes JD, Sun Z, Othieno FA, Wei BL, Wege AK (2008). Antiretroviral pre-exposure prophylaxis prevents vaginal transmission of HIV-1 in humanized BLT mice. PLoS Med.

[95] Petkova SB, Akilesh S, Sproule TJ, Christianson GJ, Khabbaz Al H, Brown AC (2006). Enhanced half-life of genetically engineered human IgG1 antibodies in a humanized FcRn mouse model: potential application in humorally mediated autoimmune disease. Int Immunol.

[96] Baxter AG, Cooke A (1993). Complement lytic activity has no role in the pathogenesis of autoimmune diabetes in NOD mice. Diabetes.

[97] Simon MA, Brodie SJ, Sasseville VG, Chalifoux LV, Desrosiers RC, Ringler DJ (1994). Immunopathogenesis of SIVmac. Virus Res.

[98] Pollom E, Dang KK, Potter EL, Gorelick RJ, Burch CL, Weeks KM (2013). Comparison of SIV and HIV-1 genomic RNA structures reveals impact of sequence evolution on conserved and non-conserved structural motifs. PLoS Pathog.

[99] Laguette N, Sobhian B, Casartelli N, Ringeard M, Chable-Bessia C, Ségéral E (2011). SAMHD1 is the dendritic- and myeloid-cell-specific HIV-1 restriction factor counteracted by Vpx. Nature.

[100] Reimann KA, Li JT, Voss G, Lekutis C, Tenner-Racz K, Racz P (1996). An env gene derived from a primary human immunodeficiency virus type 1 isolate confers high in vivo replicative capacity to a chimeric simian/human immunodeficiency virus in rhesus monkeys. J Virol.

[101] Shingai M, Donau OK, Plishka RJ, Buckler-White A, Mascola JR, Nabel GJ (2014). Passive transfer of modest titers of potent and broadly neutralizing anti-HIV monoclonal antibodies block SHIV infection in macaques. J Exp Med.

[102] Julg B, Pegu A, Abbink P, Liu J, Brinkman A, Molloy K (2017). Virological control by the CD4-binding site antibody N6 in simian-human immunodeficiency virus-infected rhesus monkeys. J Virol.

[103] Li H, Wang S, Kong R, Ding W, Lee F-H, Parker Z (2016). Envelope residue 375 substitutions in simian-human immunodeficiency viruses enhance CD4 binding and replication in rhesus macaques. Proc Natl Acad Sci USA.

[104] Saunders KO, Pegu A, Georgiev IS, Zeng M, Joyce MG, Yang Z-Y (2015). Sustained delivery of a broadly neutralizing antibody in nonhuman primates confers long-term protection against simian/human immunodeficiency virus infection. J Virol.

[105] Martinez-Navio JM, Fuchs SP, Pedreño-López S, Rakasz EG, Gao G, Desrosiers RC (2016). Host anti-antibody responses following adeno-associated virus-mediated delivery of antibodies against hiv and siv in rhesus monkeys. Mol Ther.

[106] Fuchs SP, Martinez-Navio JM, Piatak M, Lifson JD, Gao G, Desrosiers RC (2015). AAV-delivered antibody mediates significant protective effects against SIVmac239 challenge in the absence of neutralizing activity. PLoS Pathog.

[107] Saunders KO, Wang L, Joyce MG, Yang Z-Y, Balazs AB, Cheng C (2015). Broadly neutralizing human immunodeficiency virus type 1 antibody gene transfer protects nonhuman primates from mucosal simian-human immunodeficiency virus infection. J Virol.

[108] Gardner MR, Kattenhorn LM, Kondur HR, von Schaewen M, Dorfman T, Chiang JJ (2015). AAV-expressed eCD4-Ig provides durable protection from multiple SHIV challenges. Nature.

[109] Walker LM, Phogat SK, Chan-Hui P-Y, Wagner D, Phung P, Goss JL (2009). Broad and potent neutralizing antibodies from an African donor reveal a new HIV-1 vaccine target. Science.

[110] Seaman MS, Janes H, Hawkins N, Grandpre LE, Devoy C, Giri A (2010). Tiered categorization of a diverse panel of HIV-1 Env pseudoviruses for assessment of neutralizing antibodies. J Virol.

[111] Keele BF, Giorgi EE, Salazar-Gonzalez JF, Decker JM, Pham KT, Salazar MG (2008). Identification and characterization of transmitted and early founder virus envelopes in primary HIV-1 infection. Proc Natl Acad Sci USA.

[112] Salazar-Gonzalez JF, Bailes E, Pham KT, Salazar MG, Guffey MB, Keele BF (2008). Deciphering human immunodeficiency virus type 1 transmission and early envelope diversification by single-genome amplification and sequencing. J Virol.

[113] Eigen M (1971). Selforganization of matter and the evolution of biological macromolecules. Naturwissenschaften.

[114] Lynch RM, Wong P, Tran L, O’Dell S, Nason MC, Li Y (2015). HIV-1 fitness cost associated with escape from the VRC01 class of CD4 binding site neutralizing antibodies. J Virol.

[115] Louie RHY, Kaczorowski KJ, Barton JP, Chakraborty AK, McKay MR (2018). Fitness landscape of the human immunodeficiency virus envelope protein that is targeted by antibodies. Proc Natl Acad Sci USA.

[116] De BP, Hackett NR, Crystal RG, Boyer JL (2008). Rapid/sustained anti-anthrax passive immunity mediated by co-administration of Ad/AAV. Mol Ther.

[117] Trkola A, Kuster H, Rusert P, Joos B, Fischer M, Leemann C (2005). Delay of HIV-1 rebound after cessation of antiretroviral therapy through passive transfer of human neutralizing antibodies. Nat Med.

[118] Mehandru S, Vcelar B, Wrin T, Stiegler G, Joos B, Mohri H (2007). Adjunctive passive immunotherapy in human immunodeficiency virus type 1-infected individuals treated with antiviral therapy during acute and early infection. J Virol.

[119] Klein F, Halper-Stromberg A, Horwitz JA, Gruell H, Scheid JF, Bournazos S (2012). HIV therapy by a combination of broadly neutralizing antibodies in humanized mice. Nature.

[120] Kong R, Louder MK, Wagh K, Bailer RT, deCamp A, Greene K (2014). Improving neutralization potency and breadth by combining broadly reactive HIV-1 antibodies targeting major neutralization epitopes. J Virol.

[121] Wagh K, Bhattacharya T, Williamson C, Robles A, Bayne M, Garrity J (2016). Optimal combinations of broadly neutralizing antibodies for prevention and treatment of HIV-1 Clade C infection. PLoS Pathog.

[122] Huang Y, Yu J, Lanzi A, Yao X, Andrews CD, Tsai L (2016). Engineered bispecific antibodies with exquisite HIV-1-neutralizing activity. Cell.

[123] Bournazos S, Gazumyan A, Seaman MS, Nussenzweig MC, Ravetch JV (2016). Bispecific anti-HIV-1 antibodies with enhanced breadth and potency. Cell.

[124] Diskin R, Klein F, Horwitz JA, Halper-Stromberg A, Sather DN, Marcovecchio PM (2013). Restricting HIV-1 pathways for escape using rationally designed anti-HIV-1 antibodies. J Exp Med.

[125] Mingozzi F, High KA (2013). Immune responses to AAV vectors: overcoming barriers to successful gene therapy. Blood.

